# Antitumoral and Antiproliferative Potential of Synthetic Derivatives of Scorpion Peptide IsCT1 in an Oral Cavity Squamous Carcinoma Model

**DOI:** 10.3390/molecules29194533

**Published:** 2024-09-24

**Authors:** Laertty Garcia de Sousa Cabral, Cyntia Silva de Oliveira, Vani Xavier Oliveira, Rosely Cabette Barbosa Alves, Jean-Luc Poyet, Durvanei Augusto Maria

**Affiliations:** 1 Faculty of Medicine, University of Sao Paulo, Sao Paulo 05508-220, Brazil; laertty.c@usp.br; 2 Laboratory of Development and Innovation, Butantan Institute, Sao Paulo 05585-000, Brazil; rosely.alves@butantan.gov.br; 3 Paulista School of Medicine, Postgraduate Program in Molecular Biology, Federal University of São Paulo, Sao Paulo 04044-020, Brazil; cyntia.oliveira@unifesp.br (C.S.d.O.); vani.junior@ufabc.edu.br (V.X.O.J.); 4 Center for Natural and Human Sciences, Federal University of ABC, Santo Andre 09280-560, Brazil; 5 INSERM UMRS976, Institut De Recherche Saint-Louis, Hôpital Saint-Louis, 75010 Paris, France; 6 Université Paris Cité, 75006 Paris, France

**Keywords:** tongue cancer, squamous cell carcinoma, synthetic peptide, anticancer peptide, apoptosis

## Abstract

The oral cavity is a frequent site for head and neck cancers, which rank as the sixth most common cancer globally, with a 5-year survival rate slightly over 50%. Current treatments are limited, and resistance to therapy remains a significant clinical obstacle. IsCT1, a membrane-active peptide derived from the venom of the scorpion *Opisthacanthus madagascariensis*, has shown antitumor effects in various cancer cell lines, including breast cancer and chronic myeloid leukemia. However, its hemolytic action limits its potential therapeutic use. This study aims to assess the antitumor and antiproliferative activities of synthetic peptides derived from IsCT1 (IsCT-P, AC-AFPK-IsCT1, AFPK-IsCT1, AC-KKK-IsCT1, and KKK-IsCT1) in the context of oral squamous cell carcinoma. We evaluated the cytotoxic effects of these peptides on tongue squamous cell carcinoma cells and normal cells, as well as their impact on cell cycle phases, the expression of proliferation markers, modulators of cell death pathways, and mitochondrial potential. Our results indicate that the IsCT1 derivatives IsCT-P and AC-AFPK-IsCT1 possess cytotoxic properties towards squamous cell carcinoma cells, reducing mitochondrial membrane potential and the proliferative index. The treatment of cancer cells with AC-AFPK-IsCT1 led to a positive modulation of pro-apoptotic markers p53 and caspases 3 and 8, a decrease in PCNA and Cyclin D1 expression, and cell cycle arrest in the S phase. Notably, contrary to the parental IsCT1 peptide, AC-AFPK-IsCT1 did not exhibit hemolytic activity or cytotoxicity towards normal cells. Therefore, AC-AFPK-IsCT1 might be a viable therapeutic option for head and neck cancer treatment.

## 1. Introduction

The oral cavity is the primary site for head and neck cancer, one of the most common types of cancers worldwide, with a higher incidence in developing countries [1]. Oral squamous cell carcinoma (SCC) accounts for over 90% of all oral cancers and is a significant cause of morbidity and mortality in head and neck cancer. Major etiological factors include alcohol and tobacco use, diet, viruses, radiation, genetic predisposition, and immunosuppression [2].

The standard clinical approach for head and neck cancer currently involves primary surgical resection, with or without post-operative adjuvant therapy [3]. Combining radiation and chemotherapy has been shown to improve tumor-free survival rates. However, frequent mutations in apoptosis or cell cycle regulator genes such as p53 are linked to cell cycle dysregulation, increased invasiveness, and resistance to chemotherapy [4,5]. Furthermore, major treatments for head and neck cancer, such as radiation, chemotherapy, or chemoradiation therapy, can result in functional deficits, including osteoradionecrosis, xerostomia, mucositis, trismus, radiation caries, dysphagia, and altered periodontal ligaments [6]. Therefore, there is an urgent need for more efficient therapies for head and neck cancer that have greater specificity for tumor cells, thereby reducing the systemic side effects associated with treatments [7].

Peptides represent a promising alternative to conventional chemotherapy as they can overcome classic treatment shortcomings [8]. Numerous studies have indeed demonstrated that peptides offer greater target specificity, high anticancer activity, fewer side effects, and efficient intracellular transport compared to chemotherapy [9]. Peptides can be designed to recognize a broad range of biological targets, such as biological membranes, enzymes, or protein–protein interactions, giving them almost unlimited therapeutical potential [10,11,12]. Peptides also have the advantage of being degraded into non-toxic metabolites (amino acids) with low tissue accumulation and generally low immunogenicity compared with biological therapies [12,13].The use of peptides to guide drug delivery to tumors has also been shown to enhance therapeutic specificity and potential and a number of peptides have demonstrated good synergy with different conventional anticancer drugs [13,14,15,16]. Consequently, peptides have emerged as promising opportunities in cancer treatment, among which many are undergoing clinical trials [17,18,19].

The IsCT1 peptide, derived from the venom of the scorpion *Opisthacanthus madagascariensis*, is a short, 13 amino acid-long α-helical antimicrobial peptide with membrane-active properties [20]. Scorpion venom is a rich source of bioactive molecules, particularly those that block ion channels [20,21]. IsCT1 has received great interest in recent years as it exhibits both potent antimicrobial activity against Gram-positive and Gram-negative bacteria and antitumor effects toward various cancer cells, including breast cancer and chronic myeloid leukemia cells [22,23,24,25,26,27]. However, IsCT1’s therapeutic potential is limited by its substantial hemolytic activity [20,28].

Synthesizing analogs of natural peptides is a promising approach to mitigate systemic toxicity while preserving or even enhancing their therapeutic potential [29]. This study aims to evaluate the antitumoral, antiproliferative, and immunomodulatory activities of synthetic peptides derived from IsCT1 (IsCT-P, AC-AFPK-IsCT1, AFPK-IsCT1, AC-KKK-IsCT1, and KKK-IsCT1) in the context of oral cavity squamous cell carcinoma.

## 2. Results

### 2.1. Physicochemical Properties of Synthetic IsCT1 Analogs

**Figure 1:**
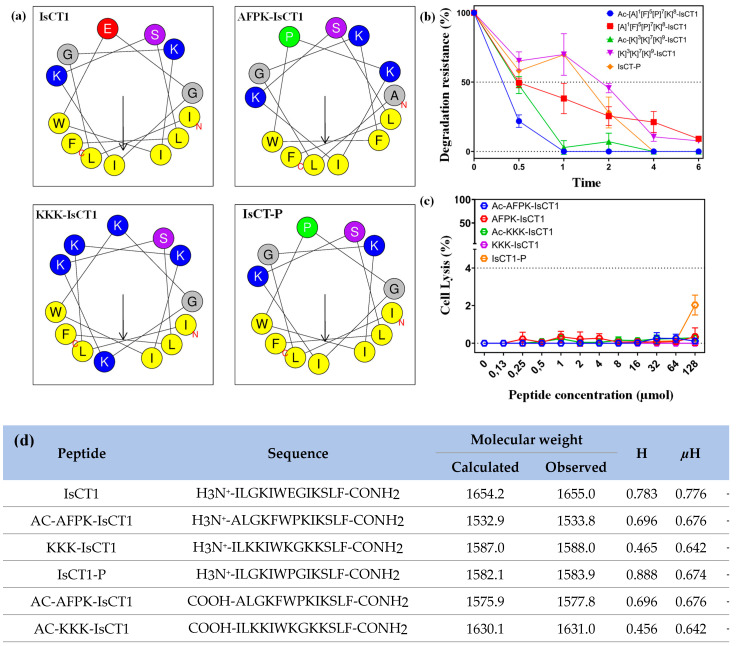
(**a**) Helical wheel projections of the IsCT1, AFPK-IsCT1, KKK-IsCT1, and IsCT-P peptides were generated using the Heliquest server (https://heliquest.ipmc.cnrs.fr/, accessed on 15 July 2024). In these projections, yellow, gray, and green circles represent hydrophobic amino acids, while blue and pink circles denote hydrophilic amino acids. (**b**) Resistance to degradation assays for all peptides were performed over 6 h in a solution containing 25% fetal bovine serum (FBS). These experiments were conducted in triplicate and aliquots were collected for analysis at 0, 0.5, 1, 2, 4, and 6 h of incubation. The aliquots were subsequently analyzed using high-performance liquid chromatography (HPLC) after each incubation period. The degradation kinetics of the peptides were determined by comparing the integrated peak area associated with the peptide at the start of the experiment (t = 0 h) with the integrated peak area at subsequent time points (t = 0.5, 1, 2, 4, 6 h). (**c**) The hemolytic activity of synthetic peptides analogous to IsCT1 was assessed on human erythrocytes within a concentration range of 0.13–128 μmol L^−1^ in phosphate-buffered saline (PBS) (*n* = 3). A 1% solution of sodium dodecyl sulfate (SDS) in PBS served as a positive control, while PBS alone was used as a negative control. (**d**) A table summarizing the sequences, molecular weights, and physicochemical properties of the IsCT1, AFPK-IsCT1, and KKK-IsCT1 peptides.

We first analyzed the physicochemical properties of the different synthesized peptides using the Heliquest server [30]. As shown in Figure 1d, the net charge analysis revealed a net positive charge of +2 for the IsCT1 peptide, which serves as the base sequence for the synthesis of the other peptide analogs. Interestingly, all the IsCT1 derivatives exhibited increased positive charges (Figure 1d) as well as an amphipathic character with a nonpolar face and a polar or charged face (Figure 1a) as observed for cationic antimicrobial peptides of the α-helical class.

Other important factors for the interaction and internalization of peptides in tumor cells are their hydrophobicity (H) and their hydrophobic moment (µM). The base peptide IsCT1 had an H of 0.783 and a µM value of 0.776, whereas the AFPK-IsCT1 peptide had an H of 0.696 and a µM value of 0.676. The KKK-IsCT1 and Ac-AFPK-IsCT1 peptides had lower H values of 0.465 and 0.456, respectively, with the same µM value of 0.642. The IsCT-P peptide had a higher H value of 0.888 and a µM value of 0.674. Finally, the Ac-AFPK-IsCT1 peptide had an H of 0.696 and a µM value of 0.676 (Figure 1d).

The antimicrobial IsCT1 peptide is fast-acting, which should alleviate the usual concern over proteolytic degradation [31]. In terms of serum stability, the AFPK-IsCT1 and KKK-IsCT1 peptides exhibited moderate stability toward proteolytic degradation, with 50% of the peptide remaining intact after 30 min of exposure to FBS and 8% after 6 h (Figure 1b). Interestingly, an analog containing a proline at position 7, IsCT-P, showed increased resistance to enzymatic degradation (Figure 1b), with 70% of the peptide remaining intact after 1 h and 18% after 4 h.

The addition of an acetyl group slightly impacted the proteolytic stability of the IsCT1 analogs. The Ac-KKK-IsCT1 analog exhibited 50% degradation in 30 min and it was completely degraded within 4 h (Figure 1b). Of note, the Ac-AFPK-IsCT1 analog exhibited greater stability, with only 18% degradation upon 30 min of exposure to the serum. The KKK-IsCT1 peptide showed a slower initial degradation curve but underwent rapid degradation after 2 h, with only 8% of the peptide remaining after 6 h (Figure 1b). These results indicate that chemical modifications or the modification of the amino acid sequence of IsCT1 induce physical–chemical changes interfering with the peptides’ stability.

Notably, none of the tested peptides, with the exception of IsCT-P, caused the hemolysis of mouse erythrocytes, which is considered a gold standard for in vitro toxicity studies (Figure 1c). This suggests that the incorporated modifications in the IsCT1 derivatives drastically reduce lytic interactions with mammalian membranes.

### 2.2. Evaluation of the Cytotoxicity of the Synthetic IsCT1 Analogs

We then assessed the efficacy of IsCT1 derivatives in tongue squamous cell carcinoma tumor SCC-9 and SCC-25 cells. As shown in Table 1, only IsCT-P and AC-AFPK-IsCT1 inhibited tumor cell viability. The calculated IC50 values for IsCT-P and AC-AFPK-IsCT1 peptides were 96.5 µM and 90.5 µM, respectively, for SCC-9 cells and 81.3 µM and 84.4 µM for SCC-25 tumor cells (Table 1). No cytotoxic effect was observed for the other tested peptides. Of note, no pronounced conformational cellular changes were observed, but there was a clear reduction in cell confluence upon treatment with either IsCT-P or AC-AFPK-IsCT1 compared to control cells (Table 1).

We next evaluated the impact of the synthesized peptides on the viability of various normal cells. As indicated in Table 1, the IsCT1P and AC-KKK-IsCT1 peptides induced cell death in FN1 fibroblasts, highlighting a lack of specificity for malignant cells. The IC50 values were 80.3 µM for the IsCT-P peptide and 100 µM for the AC-KKK-IsCT1 peptide (Table 1). Notably, the other peptides, including the tumor-active AC-AFPK-IsCT1, did not exhibit cytotoxic effects on FN1 cells (Table 1). Furthermore, none of the tested peptides showed cell death activity in normal J774 macrophage cells (Table 1). 

Combined, these data indicate that among the tested peptides, AC-AFPK-IsCT1 is the only peptide selectively cytotoxic to tongue squamous cell carcinoma tumor cells but not to normal fibroblast or macrophage cells.

### 2.3. Impact of the Synthetic IsCT1 Analogs on the Proliferative Activity of Tongue Squamous Cell Carcinoma and Normal Cells

**Figure 2:**
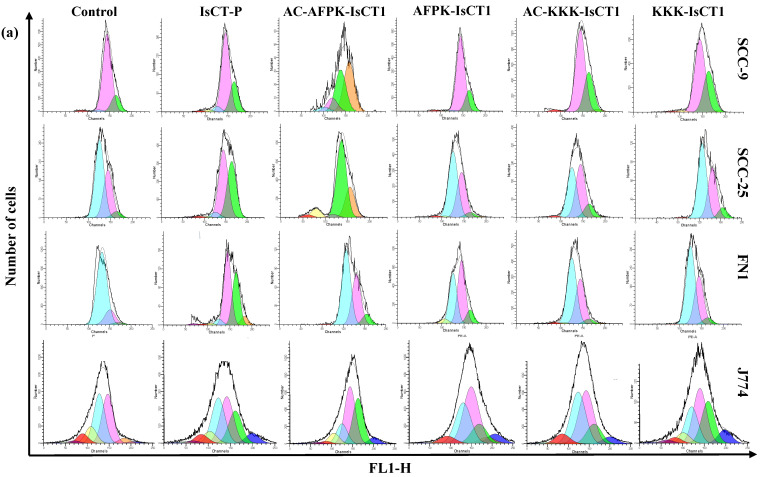

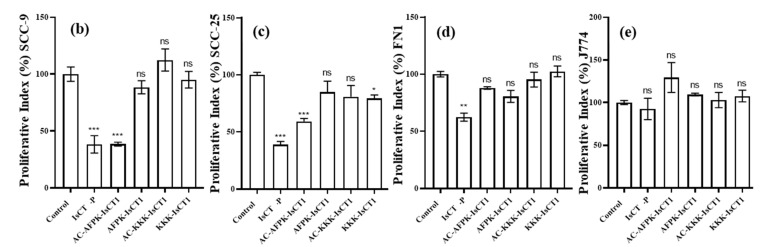
Assessment of the cellular proliferative index was conducted using flow cytometry following a 24 h treatment with synthetic analogs of the IsCT1 peptide. The treatments were performed using the IC50 values obtained for the IsCT-P and AC-AFPK-IsCT1 peptides and at a concentration of 100 µM for the other peptides. (**a**) Representative histograms of the proliferative index were analyzed using WinMDI 5.0 software. Each color in the histogram represents a new cell population. (**b**) Bar graphs represent the mean ± SD of the proliferative index for SCC-9 tumor cells derived from three independent experiments. (**c**) Bar graphs display the mean ± SD of the proliferative index for SCC-25 tumor cells based on three independent experiments. (**d**) Bar graphs represent the mean ± SD of the proliferative index for normal FN1 cells from three independent experiments. (**e**) Bar graphs illustrate the mean ± SD of the proliferative index for normal J774 cells from three independent experiments. Statistical differences were determined using ANOVA and Tukey–Kramer multiple comparison tests. Significance levels are indicated as * *p* < 0.05, ** *p* < 0.01, and *** *p* < 0.001, with ‘ns’ denoting not significant.

We further assessed the antiproliferative activity of the synthesized peptides on squamous cell carcinoma cancer cells and normal cells. In line with our previous results, a decrease in the proliferative index was noted in SCC-9 tumor cells after a 24 h treatment with the IsCT-P and AC-AFPK-IsCT1 peptides (Figure 2b). Specifically, treatment with the IsCT-P and AC-AFPK-IsCT1 peptides at IC50 concentrations resulted in a reduction of 61.8 ± 8.7% and 61.6 ± 1.7%, respectively, in the proliferative index, while no statistically significant change was observed with the other peptides (Figure 2b). A similar trend was observed with the SCC-25 tumor cells, where treatment with the IsCT-P and AC-AFPK-IsCT1 peptides at IC50 concentrations led to a decrease in the proliferative index of 61.1 ± 2.8% and 40.9 ± 2.5%, respectively, while the other IsCT1 derivatives had no effect (Figure 2c). 

The effect of peptide treatments on the proliferative index was also examined in normal human fibroblast cells. As shown in Figure 2d, only the IsCT-P peptide induced a decrease in the proliferative index, with a reduction of 37.6 ± 3.9%. No reduction in normal human fibroblast cell proliferation was observed with the other peptides (Figure 2d) or in J774 cells for all tested compounds (Figure 2e). These results show that the AC-AFPK-IsCT1 derivative possesses cancer cell specificity, whereas the IsCT-P derivative does not.

### 2.4. Impact of the Synthetic IsCT1 Analogs on Cell Cycle Phase Distribution Profile

**Figure 3:**
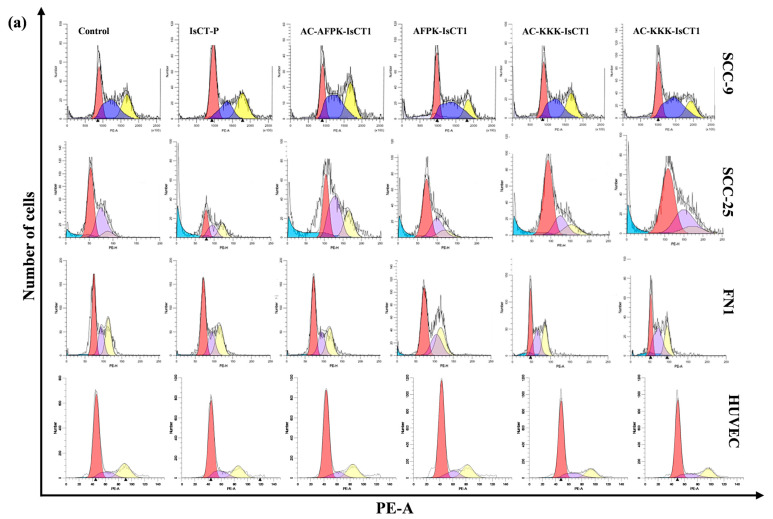

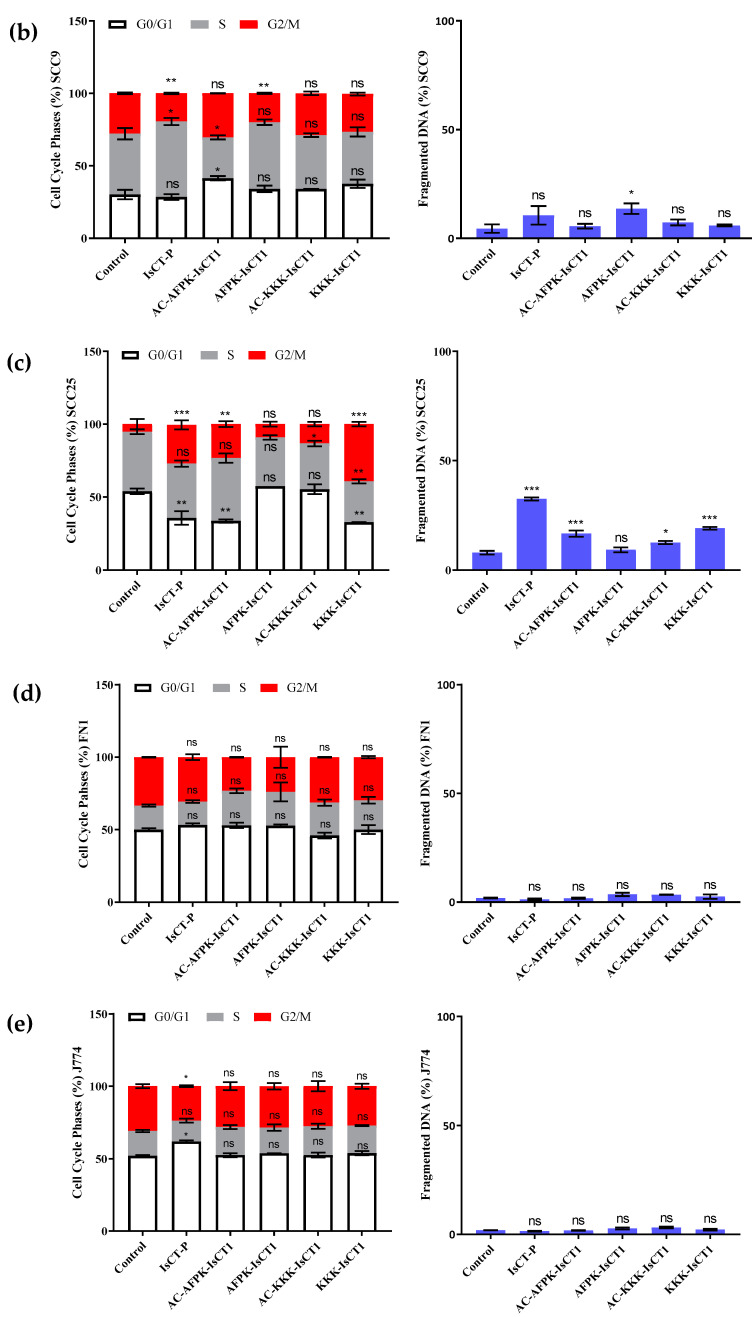
Analysis of cell cycle phases following a 24 h treatment with synthetic analogs of IsCT1 peptides. The indicated cells were treated at the IC50 values obtained for the IsCT-P and AC-AFPK-IsCT1 peptides and at a concentration of 100 µM for the other peptides. (**a**) Representative histograms of cell distribution across cell cycle phases; (**b**) bar graph showing the correlation of the effect on cell cycle and fragmented DNA, expressed as the mean ± SD of three independent SCC-9 tumor cell experiments; (**c**) bar graph showing the correlation of the effect on cell cycle and fragmented DNA, expressed as the mean ± SD of three independent experiments on SCC-25 tumor cells; (**d**) bar graph showing the correlation of the effect on cell cycle and fragmented DNA, expressed as the mean ± SD of three independent experiments of normal FN1 cells; (**e**) bar graph showing the correlation of the effect on cell cycle and fragmented DNA, expressed as the mean ± SD of three independent experiments of normal J774 cells. Statistical differences were obtained using ANOVA and Tukey–Kramer multiple comparison tests. * *p* < 0.05, ** *p* < 0.01, and *** *p* < 0.001 denote significance. ns = not significant.

We further investigated the impact of the IsCT1 derivatives on the cell cycle. In SCC-9 tumor cells, the analysis of cell cycle phase distribution revealed an arrest in the S phase (52% of cells) upon treatment with the IsCT-P peptide at IC50 concentration, together with a reduction in cells in the G2/M phase (19.4% of cells) (Figure 3a). Treatment with the AC-AFPK-IsCT1 peptide resulted in an arrest in the G0/G1 phase (42.9% of cells) and a decrease in the S phase (28.1% of cells). The AFPK-IsCT1 peptide treatment led to a reduction in the G2/M phase (19.9% of cells) without affecting the other phases of the cell cycle (Figure 3a). The control group exhibited 30.1% of cells in the G0/G1 phase, 41.9% in the S phase, and 27.8% in the G2/M phase (Figure 3a). The capacity of the peptides to induce DNA fragmentation was then assessed, with only the AFPK-IsCT1 peptide causing an increase in fragmented DNA (13.6% compared to the control) (Figure 3b).

Peptide treatments also induced significant differences in the distribution of cell populations across the cell cycle phases in SSC-25 tumor cells. Treatment with the IsCT-P, AC-AFPK-IsCT1, and KKK-IsCT1 peptides resulted in a notable arrest in the G2/M phase (26.6%, 23.3%, and 39.2% of cells, respectively) compared to the control group (5.2% of cells). A marked reduction in the G0/G1 phase population was observed upon treatment with the IsCT-P, AC-AFPK-IsCT1, and KKK-IsCT1 peptides (35.5%, 33.5%, and 32.7% of cells, respectively) compared to the control group (53.8% of cells) (Figure 3c).

We next evaluated the impact of the peptides on DNA fragmentation in SSC-25 tumor cells. As shown in Figure 3c, treatment with the IsCT-P or the AC-AFPK-IsCT1 peptides resulted in DNA fragmentation (32.4% and 16.5% fragmented DNA, respectively). No significant DNA fragmentation was observed with the AFPK-IsCT1 peptide treatment, and only a slight increase in DNA fragmentation was noted for the KKK-IsCT1 peptide and its acetylated analog AC-KKK-IsCT1 (19% and 12.4% fragmented DNA, respectively) (Figure 3c).

The effects of the peptides on the cell cycle distribution and DNA fragmentation in normal human fibroblasts (FN1 cells) were also evaluated. Interestingly, no alterations in cell cycle distribution or significant DNA fragmentation were observed with any of the peptides (Figure 3d). Similar results were obtained with normal J774 cells, except for the IsCT-P peptide treatment, which induced an arrest in the G0/G1 phase of the cell cycle (61.2% of cells) (Figure 3e). 

### 2.5. Impact of the Synthetic IsCT1 Analogs on Mitochondrial Membrane Electrical Potential (ΔΨm)

**Figure 4:**
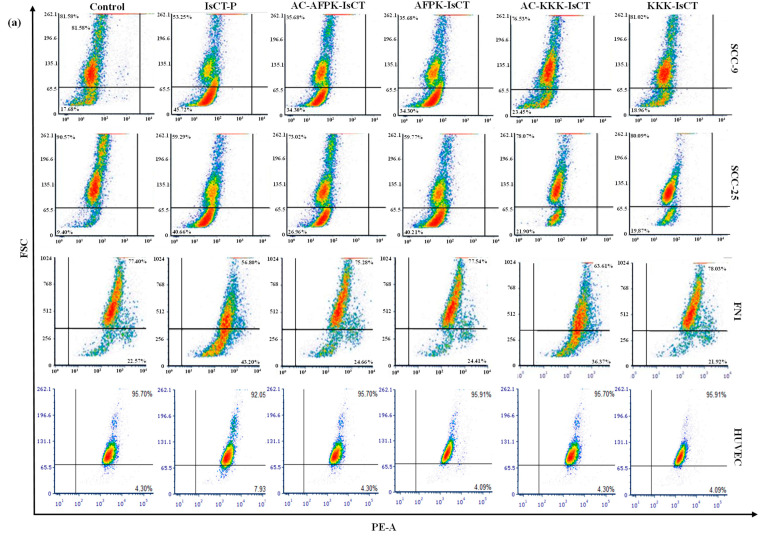

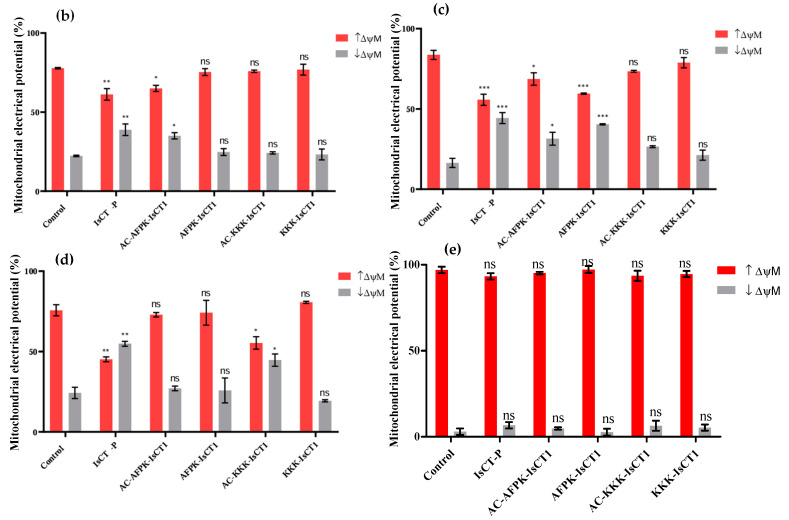
Analysis of mitochondrial electrical potential (ΔΨm) using flow cytometry following a 24 h treatment with synthetic analogs of IsCT1 peptides. The indicated cells were treated at the IC50 values obtained for the IsCT-P and AC-AFPK-IsCT1 peptides and at a concentration of 100 µM for the other peptides. (**a**) Representative histograms of cell distribution according to ΔΨm; (**b**) bar graph showing ΔΨm expressed as the mean ± SD of three independent experiments of SCC-9 tumor cells; (**c**) bar graph showing ΔΨm expressed as the mean ± SD of three independent experiments of SCC-25 tumor cells; (**d**) bar graph showing ΔΨm expressed as the mean ± SD of three independent experiments of normal FN1 cells; (**e**) bar graph showing ΔΨm expressed as the mean ± SD of three independent experiments of normal J774 cells. Statistical differences were obtained using ANOVA and Tukey–Kramer multiple comparison tests. * *p* < 0.05, ** *p* < 0.01, and *** *p* < 0.001 denote significance. ns = not significant.

We further explored the effects of the different peptides on the mitochondrial membrane electrical potential (ΔΨm) in SCC-9 tumor cells. After 24 h of treatment, a decrease in ΔΨm was noted for the IsCT-P and AC-AFPK-IsCT1 peptides, with reductions of 21.2 ± 5.1% and 16.3 ± 2.7%, respectively (Figure 4b). In line with our previous results, no significant changes were detected upon treatment with the other peptides (Figure 4b).

Notably, SCC-25 tumor cells demonstrated a greater sensitivity to the peptides in terms of mitochondrial membrane electrical potential reduction compared to SCC-9 cells. Indeed, treatment with the IsCT-P peptide resulted in a 44.4 ± 4.8% decrease in ΔΨm, while the AC-AFPK-IsCT1 peptide led to a 31.5 ± 5.4% reduction. The amidated AFPK-IsCT1 peptide also decreased ΔΨm in SCC-25 tumor cells by 40.5 ± 0.4% (Figure 4c).

We then assess mitochondrial electrical potential changes in normal FN1 cells upon peptide treatment. A reduction in ΔΨm was observed for the IsCT-P and AC-KKK-IsCT1 peptides, with decreases of 54.9 ± 2.1% and 44.7 ± 7.4%, respectively, compared to control cells (Figure 4d). No changes in ΔΨm were observed for the other peptides, including AC-AFPK-IsCT1. Lastly, no statistically significant alterations in the mitochondrial electrical potential were observed in normal J774 cells with any of the peptides (Figure 4e).

**Figure 5:**
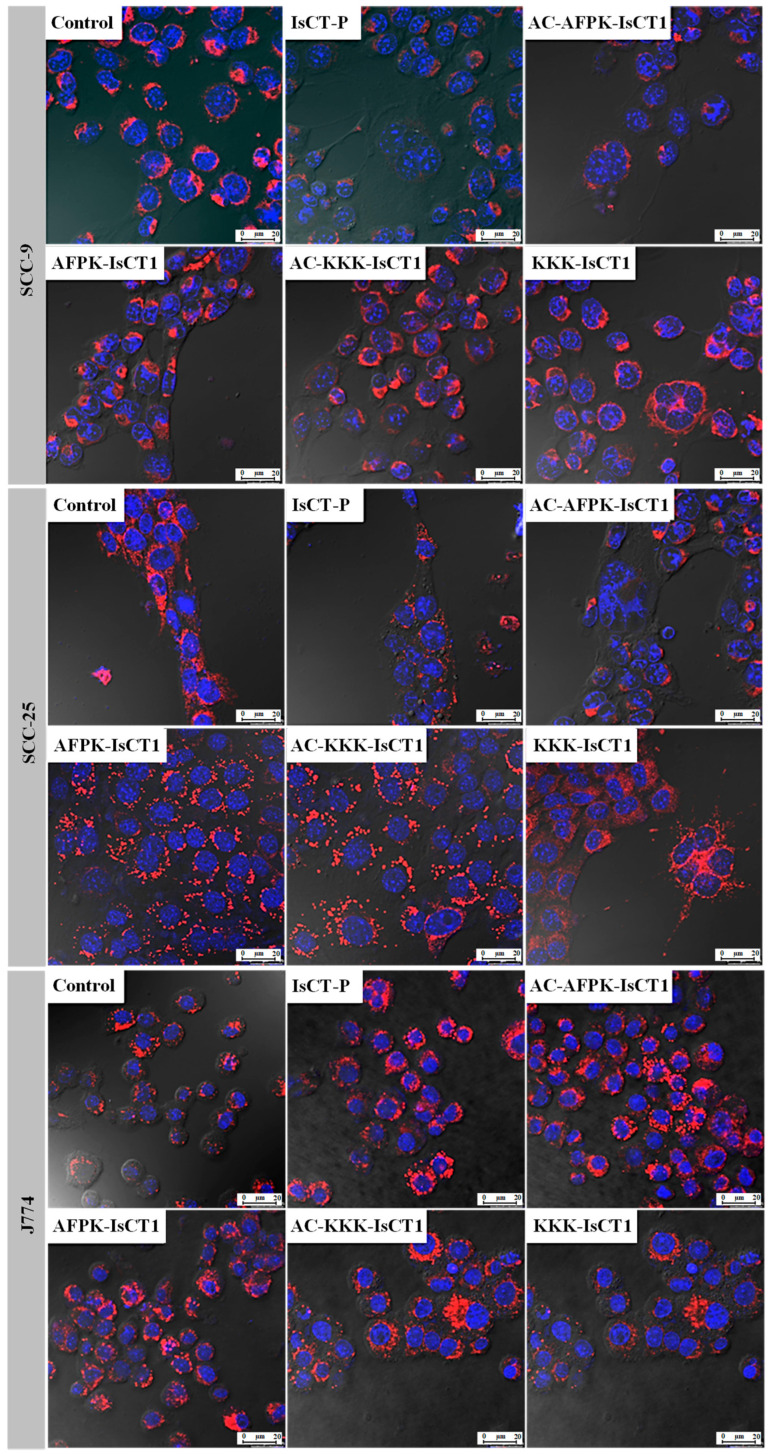
Photomicrographs of tongue squamous cell carcinoma tumor cells (SCC-9 and SCC-25) and macrophages (J774), with mitochondria stained red (MitoRED) and nuclei stained blue (DAPI), analyzed using confocal laser microscopy. The indicated cells were treated at the IC50 values obtained for the IsCT-P and AC-AFPK-IsCT1 peptides and at a concentration of 100 µM for the other peptides for a period of 24 h.

We next determined mitochondrial potential changes using confocal microscopy by loading mitochondria with MitoRed and staining nuclei with DAPI. These data are crucial for assessing the peptides’ ability to induce cell death or disrupt vital structures involved in regulated cell death. In line with the results obtained from the flow cytometry analysis, peptides IsCT-P and AC-AFPK-IsCT1 promoted a reduction in the mitochondrial electric potential in the tumor cells SCC-9 and SCC-25 (Figure 5). On the other hand, the other peptides, at the treatment concentrations used, did not demonstrate cytotoxic potential (Figure 5). No significant changes were observed in the modulation of fluorescence in J774 cells, suggesting that the peptides do not exhibit cytotoxicity for normal macrophages (Figure 5).

Overall, our results indicate that among the IsCT1 derivatives, only AC-AFPK-IsCT1 exhibits anticancer effects while sparing normal cells.

### 2.6. Effect of the AC-AFPK-IsCT1 Peptide on the Expression of Proteins Involved in Cell Death and Proliferation in Tumor Cells

**Figure 6:**
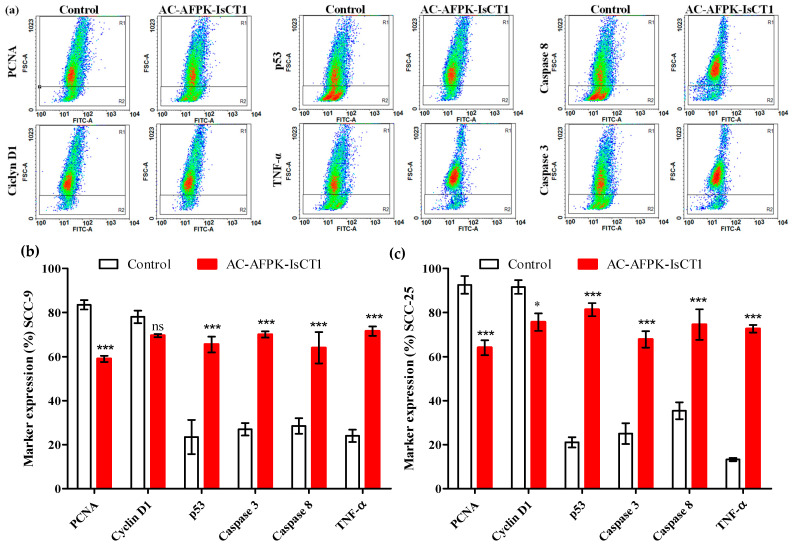
Analysis of marker expression in tongue squamous cell carcinoma tumor cells. The expression of markers was quantified using flow cytometry after 24 h of treatment with the AC-AFPK-IsCT1 peptide at its IC50 value determined for each cell line. (**a**) Representative density plots display the distribution of cells with fluorescence intensity; (**b**) marker expression in SCC-9 tumor cells; (**c**) marker expression in SCC-25 tumor cells. Bar graphs present protein expression levels as the mean ± SD from three independent experiments. Values are expressed as the mean ± SD from three independent experiments. Statistical differences were determined using ANOVA and Tukey–Kramer multiple comparison tests. * *p* < 0.05 and *** *p* < 0.001 denote significance. ns = not significant.

We subsequently investigated the impact of the AC-AFPK-IsCT1 peptide on the expression patterns of proteins related to cell death and cell proliferation. For that purpose, SCC-9 and SCC-25 tumor cells were treated for 24 h with AC-AFPK-IsCT1 at the IC50 concentrations determined for each cell line and the expression of various regulators of cell death and cell cycle progression was assessed using flow cytometry. In SCC-9 tumor cells, AC-AFPK-IsCT1 peptide treatment resulted in a significant decrease in the expression of the proliferation marker PCNA, with a reduction of 21.4 ± 0.4% compared to control cells (Figure 6b). No significant changes were noted in the expression of Cyclin-D1 after peptide treatment (Figure 6b).

Importantly, a marked increase in the expression of cell death markers was observed in SCC-9 cells exposed to AC-AFPK-IsCT1. Indeed, treatment with the AC-AFPK-IsCT1 peptide led to a 60.7 ± 1.6% increase in cells expressing p53 and a 69.2 ± 2.6% increase in cells expressing tumor necrosis factor-alpha (TNF-α) compared to control cells (Figure 6b). Additionally, the expression of caspases 3 and 8 was elevated by 67 ± 4.9% and 61.6 ± 3%, respectively, upon treatment with the AC-AFPK-IsCT1 peptide compared to control cells (Figure 6b).

Similar findings were observed in SCC-25 tumor cells, where treatment with the AC-AFPK-IsCT1 peptide resulted in a significant reduction in cell proliferation markers and an increase in cell death markers. Compared to control cells, the expression of PCNA and Cyclin D1 decreased by 22.8 ± 2.5% and 15 ± 1.3%, respectively, following AC-AFPK-IsCT1 peptide treatment (Figure 6c). A substantial increase in the expression of the pro-apoptotic protein p53 was also observed, with an 83.5 ± 1.8% increase in p53-expressing cells. Furthermore, treatment with the AC-AFPK-IsCT1 peptide induced an 85.6 ± 8.4% increase in TNF-α-expressing cells (Figure 6c). There was also a substantial elevation in the expression of caspases 3 and 8, with increases of 86.2 ± 7% and 82.3 ± 5.5%, respectively, following treatment with the AC-AFPK-IsCT1 peptide (Figure 6c).

Combined, our data indicate that the AC-AFPK-IsCT1 peptide can alter gene expression in tumor cells, downregulating cell cycle positive regulators and antiapoptotic genes and favoring the induction of proapoptotic genes.

## 3. Discussion

In this study, we evaluated the efficacy of synthetic analogs of the IsCT1 peptide, derived from the venom of *O. madagascariensis*, in inhibiting cell proliferation and inducing cell death in tongue squamous cell carcinoma cells. Among the synthesized IsCT1 derivatives, the AC-AFPK-IsCT1 peptide exhibited great specificity for tumor cells while showing no cytotoxicity toward normal FN1 and J774 cells. Moreover, contrary to the parental IsCT1, AC-AFPK-IsCT1 did not show hemolytic activity at high concentrations, indicating that the introduced modifications minimize hemolytic activity and the cytotoxic effect of IsCT1 on normal cells while maintaining its antitumoral properties. Exposure of squamous cell carcinoma cells to the AC-AFPK-IsCT1 peptide altered the expression of proteins involved in cell death and cell progression, together with a reduction in the proliferative index and a decrease in mitochondrial membrane potential, leading to cell cycle arrest at the G0/G1 and S phases. These results suggest the potential of this peptide to modulate senescence pathways and selectively regulate cell death in this type of cancer while sparing normal cells.

The efficacy of antitumor drugs is often limited by a lack of specificity and satisfactory bioavailability in the tumor microenvironment, as well as the systemic side effects that are associated with traditional chemotherapy. Cisplatin is one of the most potent chemotherapeutic agents currently used to treat oral cavity cancers, exerting its cytotoxic action through the formation of intra-strand DNA cross-links. However, the therapeutic benefits resulting from cisplatin-induced DNA damage can be mitigated, and subsequent resistance is a major limitation of cisplatin-based chemotherapy [32,33]. The molecular mechanisms underlying cisplatin resistance are not fully understood and may vary between different tumor types [34]. It is believed that the molecular signature defining the cisplatin-resistant phenotype differs among tumors and generally involves multiple factors [35]. Sixty-three genes related to cisplatin resistance have indeed been identified, including the decreased expression of cell cycle checkpoint genes and the increased expression of oncogenes, cell cycle regulatory genes, and genes involved in metabolism and synthesis [36]. These alterations lead to an accelerated cell cycle, increased proliferation, and resistance to apoptosis. Among them, the CCND1 and CCND3 genes appear to be importantly involved in cisplatin resistance [37].

Protein- and peptide-based therapies offer unique advantages, including high pharmacological potency, molecular specificity, multifunctionality, and low toxicity, making them promising alternatives for cancer therapy [38,39]. Scorpions, which are venomous arthropods, use their venom for hunting and protection. Scorpion venom contains various bioactive molecules, including a number of antimicrobial peptides. These peptides can be categorized into two classes, namely those with disulfide bridges, typically targeting ion channels on cell membranes, and those without disulfide bridges, which have various mechanisms of action.

IsCT1 is a non-disulfide bridge antimicrobial peptide isolated from *Opisthacanthus madagascariensis* scorpion venom. It has shown activity against both Gram-positive and Gram-negative bacteria as well as substantial hemolytic activity [20] which precludes its clinical use. Other analogs of IsCT1 have been synthesized, demonstrating that alterations in the peptide’s hydrophilic region affect its biological activity [22,24,40,41].

Cationic cell-penetrating peptides exhibit antiproliferative properties through diverse targets, including the cell nucleus and mitochondria, triggering regulated and unregulated cell death with a high specificity for tumor cells [42,43]. The selective cytotoxicity of cationic peptides toward tumor cells may be attributed to the increased electronegativity of tumor cell membranes due to the heightened expression of anionic elements such as heparan sulfate, phosphatidylserine, and sialic acid [42]. Tumor cell membranes also feature an increased presence of microvilli, enhancing the peptides’ uptake [42].

It has been reported that the venom of the scorpion *Androctonus amoreuxi* possesses cytotoxic and antiproliferative properties against human prostate cancer cells (PC3) [44]. These effects are driven by the negative regulation of the antiapoptotic gene Bcl-2 at the RNA level and an increase in the Bax/Bcl-2 ratio, leading to the release of cytochrome c from the mitochondria to the cytosol and the induction of apoptosis [44]. Our data indicate that the AC-AFPK-IsCT1 peptide promoted the regulated cell death of tumor cells through the modulation of the tumor suppressor protein p53, as well as caspases 8 and 3. Moreover, AC-AFPK-IsCT1 treatment led to a reduction in the expression of the proliferation markers PCNA and Cyclin D1, resulting in a significant reduction in tumor cell proliferation as well as a decrease in mitochondrial membrane potential and an elevation in TNF-α expression without affecting normal cells. Therefore, AC-AFPK-IsCT1 might be a viable therapeutic option for head and neck cancer treatment.

Further studies will have to be conducted to better understand the mechanisms underlying the cytotoxic effect of the AC-AFPK-IsCT1 peptide and evaluate the benefit of its combination with chemotherapeutic drugs, such as cisplatin, in appropriate murine models. Nevertheless, our study demonstrates that our approach, based on the chemical modification of natural peptides, can lead to the development of cancer cell-specific peptides with reduced cytotoxicity.

## 4. Materials and Methods

### 4.1. Solid-Phase Peptide Synthesis (SPPS), Purification, and Analysis

The peptides IsCT (ILGKIWEGIKSLF), IsCT-P (H_3_N^+^-ILKKIWKPIKKLF-CONH_2_, AC-AFPK-IscT1 (COOH-ALGKFWPKIKSLF-CONH_2_), AFPK-IsCT1 (H_3_N^+^-ALGKFWPKIKSLF-CONH_2_), AC-KKK-IsCT1 (COOH-ILKKIWKGKKSLF-CONH_2_), and KKK-IsCT1 (H_3_N^+^-ALGKFWPKIKSLF-CONH_2_) were synthesized using a peptide synthesizer (PS3-SyncTechnologies) employing the solid-phase synthesis methodology and the fluoromethylcarbonil (Fmoc) strategy, as detailed by Torres et al. (2017) [45]. Purification assays were conducted through semi-preparative reverse-phase high-performance liquid chromatography (RP-HPLC) on a Delta Prep 600 (Waters Associates, Milford, MA, USA). Selected fractions containing the purified peptides were collected and lyophilized. Purity was assessed using an Alliance HPLC system (Waters Associates) (Figure S1) and characterized using liquid chromatography/electrospray ionization mass spectrometry (LC/ESI-MS) with a 6130 Infinity mass spectrometer coupled to a 1260 HPLC system (Agilent, Santa Clara, CA, USA) (Figure S2).

### 4.2. Peptide Stability Assays

Stability assays were conducted using GIBCO (Waltham, MA, USA) fetal bovine serum diluted to 25% in water, as described by Torres et al. [46]. Briefly, peptide solutions were added to the serum solution and maintained at 37 °C. The experiments were carried out in triplicate and samples were taken at 0, 0.5, 1, 2, 4, and 6 h post incubation. The degradation kinetics were monitored using liquid chromatography and the percentage of remaining peptides was calculated by integrating the peak area of the peptide.

### 4.3. Cell Culture

The cell lines used for oral cavity squamous carcinoma, namely SSC-9 (ATCC^®^ CRL-1629™), SCC-25 (ATCC^®^ CCRL-1628™), and J-774 (ATCC^®^ TIB-67™), were obtained from the American Type Culture Collection (Manassas, VA, USA). The normal human fibroblast cell line FN1 (CAPPesq HCFMUSP No. 921/06) was isolated by Professor Dr. Durvanei Augusto Maria. SCC-9 and SCC-25 cells were cultured in a 1:1 mixture of DMEM (Cultilab, Campinas, SP, Brazil) and Ham’s F12 medium (Cultilab, Campinas, SP, Brazil), supplemented with 1.2 g/L sodium bicarbonate, 2.5 mM L-glutamine, 15 mM HEPES, 0.5 mM sodium pyruvate, 400 ng/mL hydrocortisone, and 10% fetal bovine serum. FN1 cells were cultured in RPMI-1640 medium (LGC Biotecnologia, Cotia, SP, Brazil) containing 10% fetal bovine serum, 100 units/mL Penicillin G, and 100 μg/mL streptomycin. Cells were incubated at 37 °C with 5% CO_2_.

### 4.4. Cell Viability and Hemolysis Assays 

Cell survival was assessed using an MTT assay. Tumor (SSC-9 and SSC-25) and normal cells (FN1 and J-774) were incubated in 96-well plates at a density of 105 cells/mL for 24 h. Subsequently, treatments were administered as described in Table 1 for 24 and 48 h. After the treatment period, the supernatant was aspirated and 100 μL of MTT at a concentration of 5 mg/mL (Calbiochem—Darmstadt, Germany) was added to the plate and incubated for 3 h at 37 °C with 5% CO_2_. Afterward, the content was removed and 200 μL of methanol was added to dissolve the formazan crystals. Absorbance was then measured at a wavelength of 540 nm using a microplate reader. The hemolysis assay was performed as previously reported [47].

### 4.5. CFSE-DA Proliferation Assay

Tumor (SSC-9 and SSC-25) and normal cells (FN1 and J-774) were plated in 24-well plates at a density of 10^5^ cells/mL for 24 h. The treated and control groups were incubated with the carboxyfluorescein marker (CFSE-DA Thermo Fisher (Waltham, MA, USA), KITC34571). CFSE-DA was diluted in PBS + 0.1% human albumin and added to the cell-containing medium. After 24 h of treatment, the cells were trypsinized, transferred to a conical tube, and centrifuged at 1500 rpm for 5 min. The supernatant was discarded and the pellet was resuspended in 1 mL of 4% paraformaldehyde in PBS for 30 min. The cells were then centrifuged, the supernatant was discarded, and the pellet was resuspended in 200 μL of FACS buffer. Readings were performed on a FACScanto flow cytometer (BD, Franklin Lakes, NJ, USA) with the number of events set to 10,000 events, and histograms were acquired and analyzed using ModFit LT 5.0 software.

### 4.6. Cell Cycle Phase and DNA Fragmentation Analysis Using Flow Cytometry

Tumor (SSC-9 and SSC-25) and normal cells (FN1 and J-774) were subjected to treatment for 24 h at the peptides’ IC50 concentrations. Treated and control cells were trypsinized and centrifuged at 1200 rpm for 5 min. The resulting pellet was resuspended in 1 mL of cold buffer, followed by the addition of 3 mL of absolute ethanol. Samples were stored at −20 °C for 24 h. The samples were then centrifuged at 1500 rpm for 10 min and resuspended in 200 μL of FACS buffer containing 0.1% Triton X-100 (Sigma-Aldrich, St. Louis, MO, USA), 50 μg/mL propidium iodide (Sigma-Aldrich), and 1 µL of RNAse (200×). This mixture was kept at room temperature and protected from light for 30 min. The samples were then transferred to cytometry tubes and analyzed using a FACScanto flow cytometer (BD) with 10,000 events, and histograms were acquired and analyzed using ModFit LT 5.0 software.

### 4.7. Mitochondrial Membrane Potential Using Flow Cytometry and Confocal Microscope Analysis

Tumor (SSC-9 and SSC-25) and normal cells (FN1 and J-774) were subjected to treatment for a period of 24 h at the peptides’ IC50 concentrations. After treatment, the cells were trypsinized, centrifuged at 1200 rpm for 5 min, the supernatant was discarded, and the cells were resuspended in complete medium containing MitoRed™ (Sigma-Aldrich, USA) (200 nM/L). Subsequently, the samples were incubated in an incubator at 37 °C with 5% CO_2_ for 30 min. The tubes were then centrifuged, the supernatant was discarded, and the pellet was resuspended in 200 μL of FACS buffer. Readings were performed using a FACScanto flow cytometer (BD) with 10,000 events, and histograms were acquired and analyzed using FCS Express software 2.0.

For the confocal microscopy ana.lysis, tumor and normal cells were cultured in 24-well plates containing round coverslips with RPMI and Leibovitz medium, respectively, supplemented with 10% FBS and incubated with 5% CO_2_ at 37 °C for 24 h. Samples from the control and treated groups were subjected to the process of removing the culture medium and washing with RPMI medium. A total of 200 nML^−1^ of MitoRed (Sigma-Aldrich, USA) was then added and the cells were incubated for 1 h in the dark at 37 °C. After incubation, the cells were washed with PBS, incubated with MitoRed, and fixed with 4% paraformaldehyde for 30 min. The cells were then washed with PBS and incubated. A total of 10 µL of VECTASHIELD^®^ antifade with DAPI (Vector Laboratories, Burlingame, CA, USA) was used on the slide to promote the sealing and marking of the nucleus. The slides were stored in a closed place until the day of reading. Coverslips were placed on slides for observation under a confocal laser fluorescence microscope (Fluoview™ 300) and images were documented and analyzed.

### 4.8. Evaluation of Cellular Marker Expression Using Flow Cytometry

Tumor cells SSC-9 and SSC-25, both treated with the peptides at their IC50 concentrations for 24 h and control groups (10^5^ cells/mL), were incubated at 4 °C for 1 h with 1 μg of specific antibodies conjugated with Alexa Fluor™ 488. Various markers associated with cell death and cell cycle progression, such as caspase 3 and 8, cyclin D1, PCNA, p53, and TNF-α conjugated with Alexa Fluor™ 488, were used as indicated. For intracellular markers, TritonX 0.1% was used for 30 min at room temperature before antibody labeling. Following centrifugation at 1500 rpm and washing with PBS, the supernatant was discarded and the pellet was resuspended in 200 μL of FACS buffer. The reading and analysis of receptor expression on the cell surface of tumor cells were carried out using a FACScanto flow cytometer (BD) with fluorescence intensity FL1-H for the number of events (10,000 events), and the dot plots were acquired and analyzed using FCS Express software.

### 4.9. Statistical Analyses

All values obtained from the different cell lines will be expressed as the mean ± standard deviation. After obtaining individual values for each treated and control cell line, the results will be tabulated and analyzed using GraphPad Prism 5.0 and 8.0 software. Data analysis will be performed by comparing three or more groups with a non-parametric distribution using analysis of variance (ANOVA) test followed by the Tukey–Kramer multiple comparison test, considering *p* < 0.05 as the critical level for significance.

## 5. Conclusions

Despite advances in diagnosis and treatment, oral squamous cell carcinoma, the most common type of head and neck cancer, exhibits a poor prognosis, highlighting the need for new treatment strategies. We presented here the design, synthesis, and characterization of synthetic analogs of the antimicrobial peptide IsCT1—found in the venom of the scorpion Opithancatus Madagascariensis—and their evaluation in the context of oral squamous cell carcinoma. IsCT1 has been previously demonstrated to possess potent anticancer properties, but its high hemolytic activity remains a challenge and prevents clinical application. Like the natural IsCT1, the synthesized analogs exhibited amphipathic helix structures. Interestingly, the analog AC-AFPK-IsCT1 exhibited promising anticancer properties toward oral squamous cell carcinoma cells, with no toxicity toward normal cells and an absence of hemolytic activity. Mechanistically, AC-AFPK-IsCT1 induces regulated cell death in tumor cells by increasing p53 and TNF-α levels, as well as caspases 8 and 3 levels, while downregulating the expression of proliferation markers PCNA and Cyclin D1. Additionally, AC-AFPK-IsCT1 decreases mitochondrial membrane potential. Evaluating structural modifications is essential for the development of new antitumoral agents and for the reduction in non-specific cytotoxic effects. Our structure–activity relationship studies have revealed several starting points for the optimization for future designs of antitumor peptides derived from IsCT1 and suggest that the analog AC-AFPK-IsCT1 might constitute an innovative therapeutic compound for oral squamous cell carcinoma treatment.

## Figures and Tables

**Table 1:** IC50 values of IsCT1 and its derivatives for tumor cells (SCC-9 and SCC-25) and normal cells (FN1 and J774).

Treatment	Cell	IC50 (µM)	Cell	IC50 (µM)
IsCT-P		96.54		81.32
AC-AFPK-IsCT1		90.50		84.48
AFPK-IsCT1	SCC-9	N/IC50	SCC-25	N/IC50
AC-KKK-IsCT1		N/IC50		N/IC50
KKK-IsCT1		N/IC50		N/IC50
IsCT-P		80.38		N/IC50
AC-AFPK-IsCT1		N/IC50		N/IC50
AFPK-IsCT1	FN1	N/IC50	J774	N/IC50
AC-KKK-IsCT1		100		N/IC50
KKK-IsCT1		N/IC50		N/IC50

N/IC50 = no IC50 for the tested concentrations; no toxicity.

## Data Availability

Data are contained within the article and Supplementary Materials.

## Supplementary Materials

The following supporting information can be downloaded at: https://www.mdpi.com/article/10.3390/molecules29194533/s1, Figure S1: LC/ESI-MS profiles of the purified synthetic peptides. Figure S2: HPLC analysis of the purified synthetic peptides.
