# Supplementary material for: Antitumoral and Antiproliferative Potential of Synthetic Derivatives of Scorpion Peptide IsCT1 in an Oral Cavity Squamous Carcinoma Model
Source: Molecules. 2024 Sep 24;29(19):4533. doi: 10.3390/molecules29194533 (PMC11478212; doi:10.3390/molecules29194533)
Supplement: Supplementary file 1 [file molecules-29-04533-s001.zip › molecules-3128559-supplementary.pdf]

Supplementary information:

Figure legends

Figure S1: LC/ESI-MS profiles of the purified synthetic peptides. (a) Mass scan of IsCT-P in 11.441min, *m/z*; **(b)** Mass scan of AFPK-IsCT in 11.810min, *m/z*; **(c)** Mass scan of Ac-AFPK-IsCT in 12.567min, *m/z*; **(d)** Mass scan of in KKK-IscT in 9.702min, *m/z*; **(e)** Mass scan of in Ac-KKK-IscT in 10.253min, *m/z*; **(f)** Table showing calculated and observed mass.

Figure S2: Analytical HPLC of the purified peptides. (a) Chromatogram of the IsCT-P peptide; (b) Chromatogram of the AFPK-IsCT peptide; (c) Chromatogram of the AC-AFPK-IsCT peptide; (d) Chromatogram of the KKK-IsCT peptide; (e) Chromatogram of the AC-AFPK-IsCT peptide; (f) Table showing the purity level obtained from the chromatogram for the samples.

Figure S1:

(a)

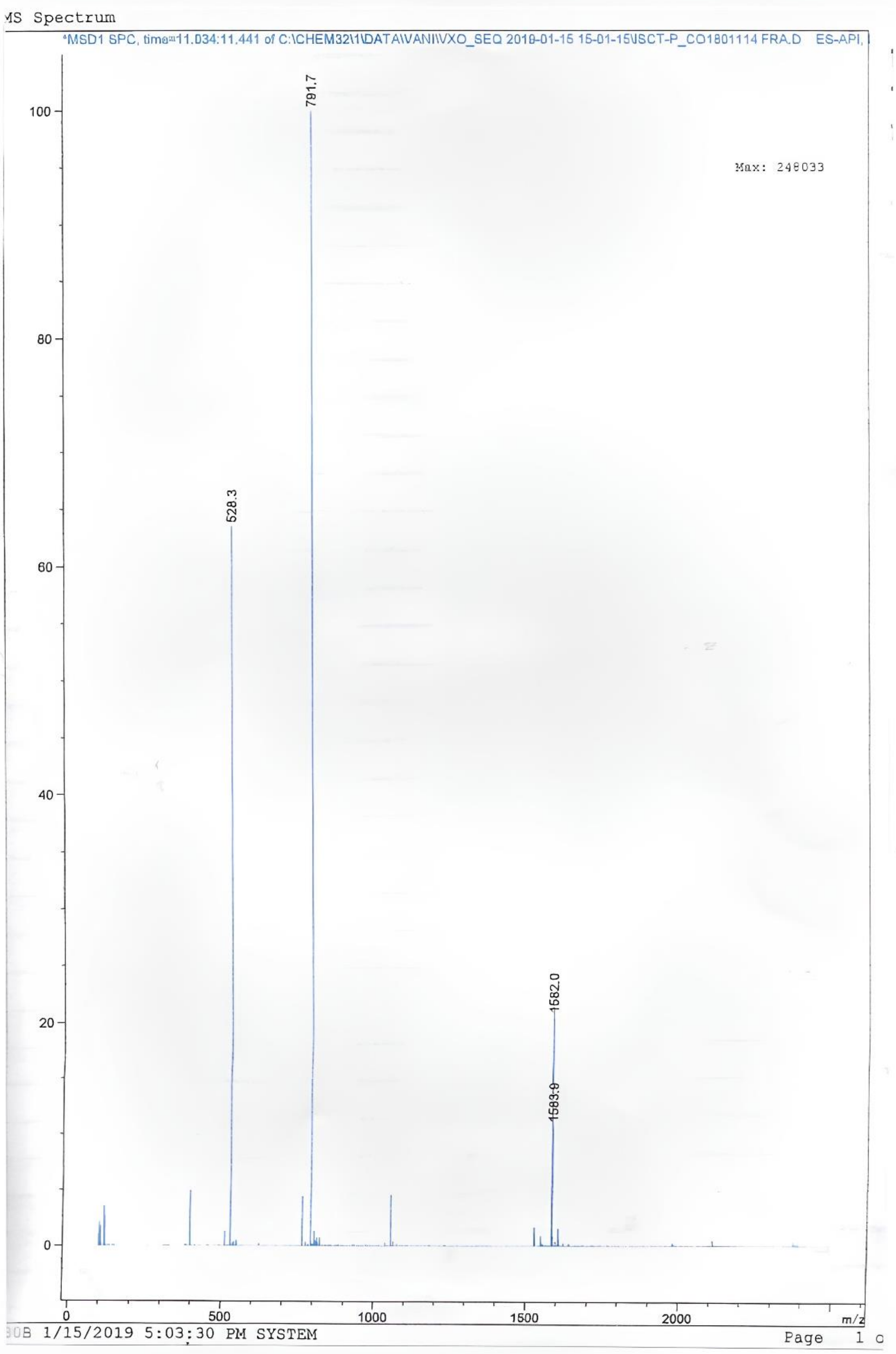
MS Spectrum
*MSD1 SPC, time=11.034:11.441 of C:\CHEM32\1\DATA\VANI\VXO_SEQ 2019-01-15 15-01-15\SCT-P_CO1801114 FRA.D ES-API,
Max: 248033
791.7
528.3
1582.0
1583.9
100
80
60
40
20
0
0
500
1000
1500
2000
m/z
1/15/2019 5:03:30 PM SYSTEM
Page 1

(b)

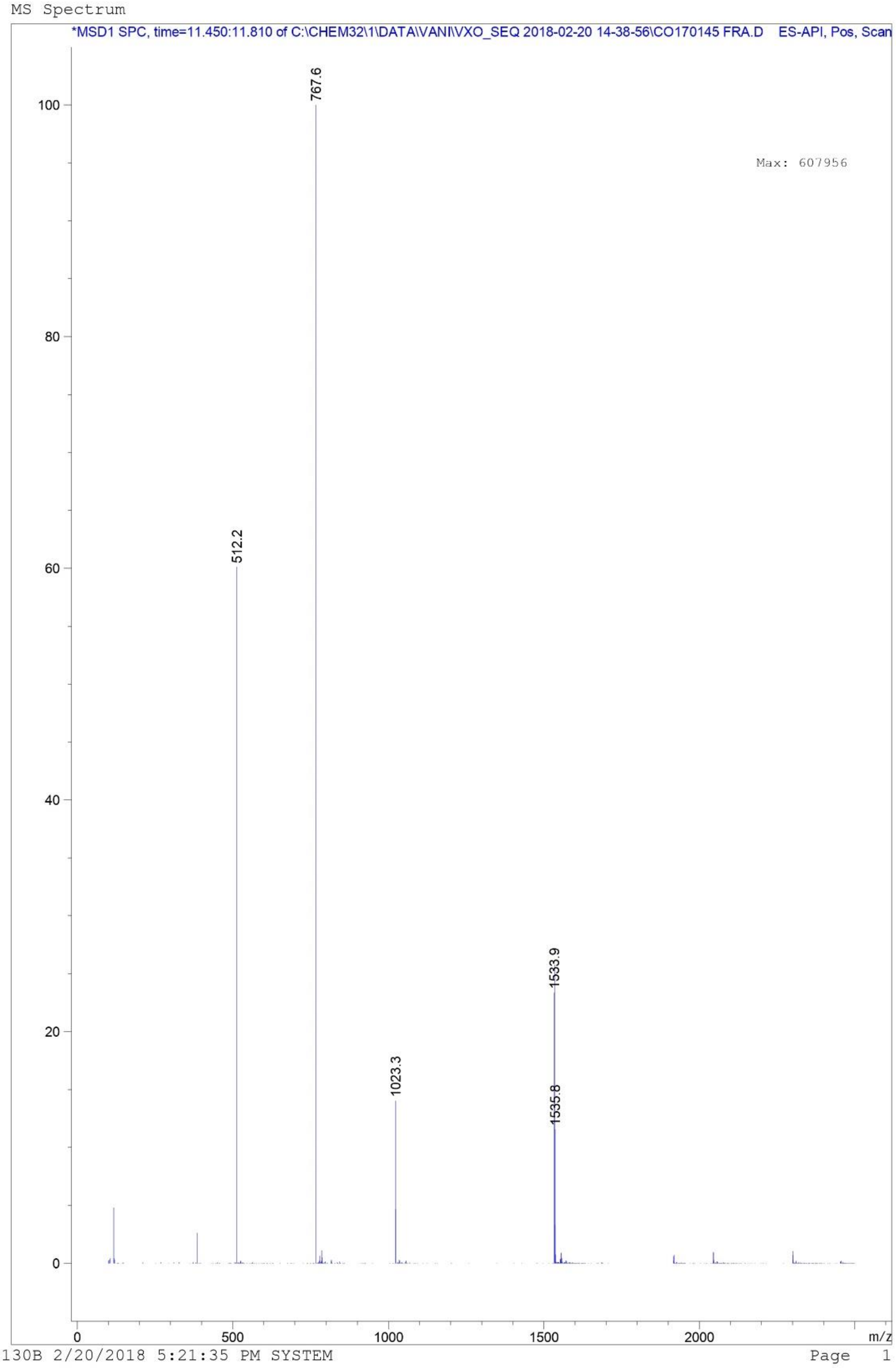

LCMS 6130B 2/20/2018 5:21:35 PM SYSTEM Page 1 of 1

Print of window 80: MS Spectrum

(c)

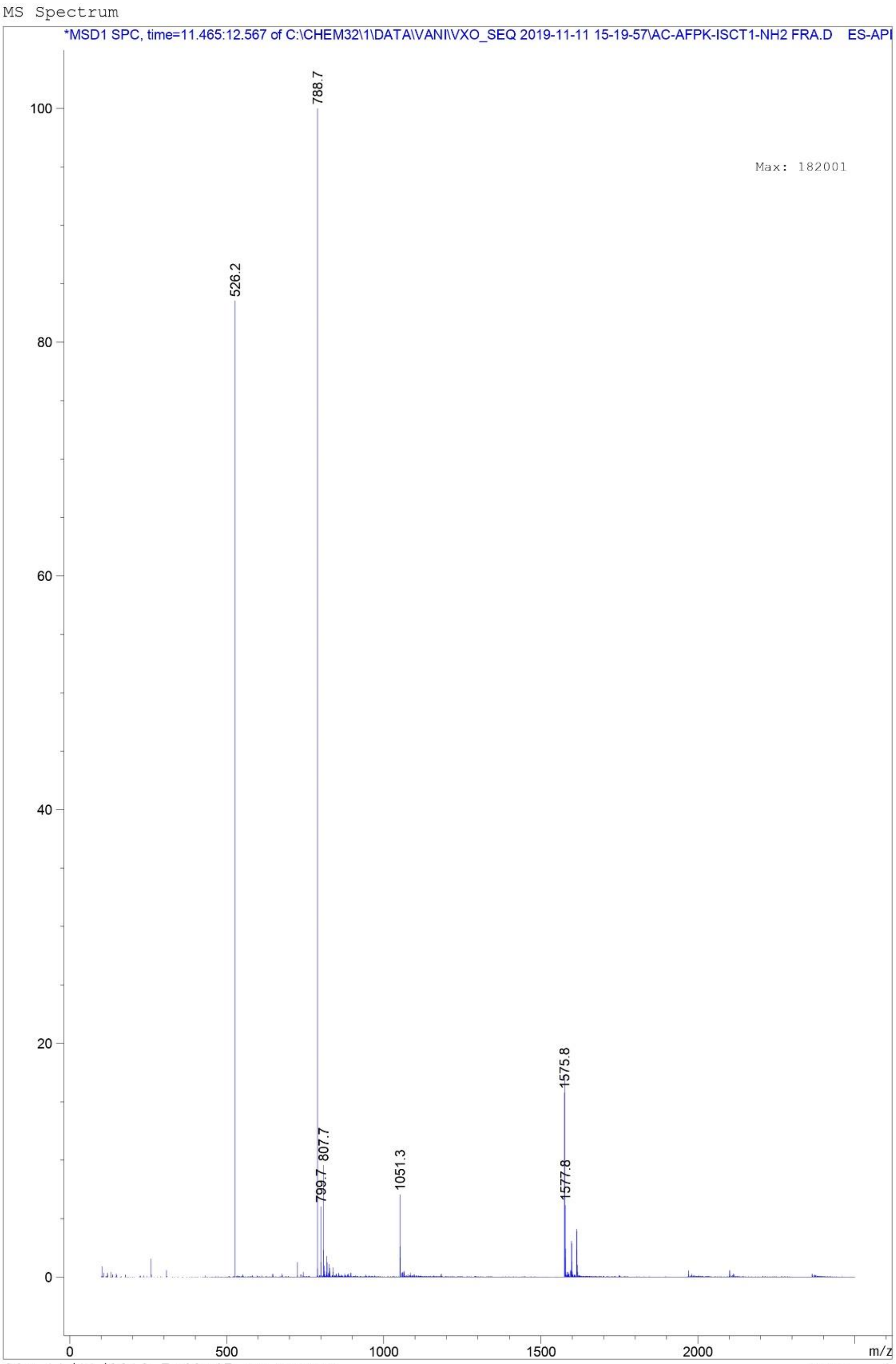

(d)

MS Spectrum

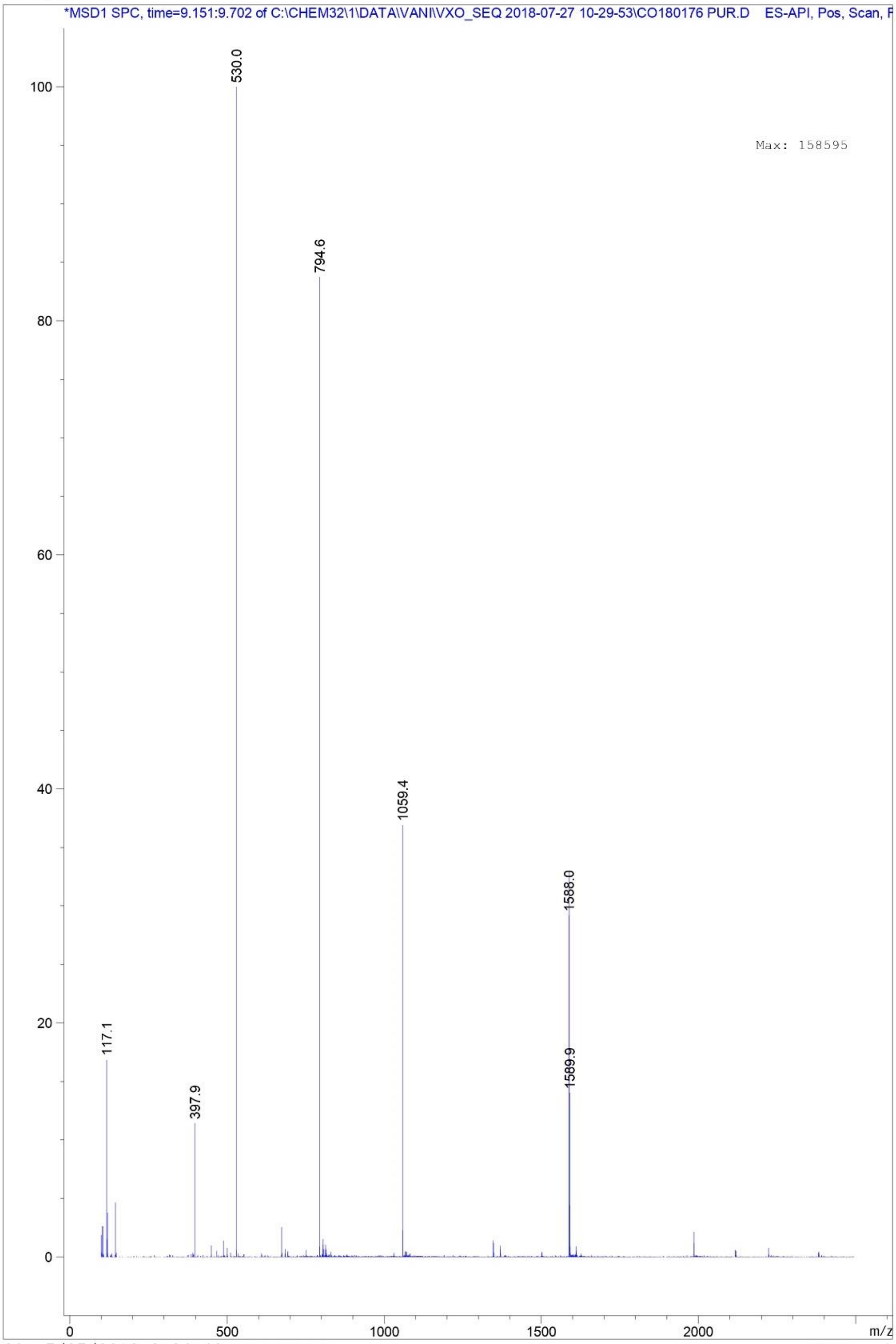

(e)

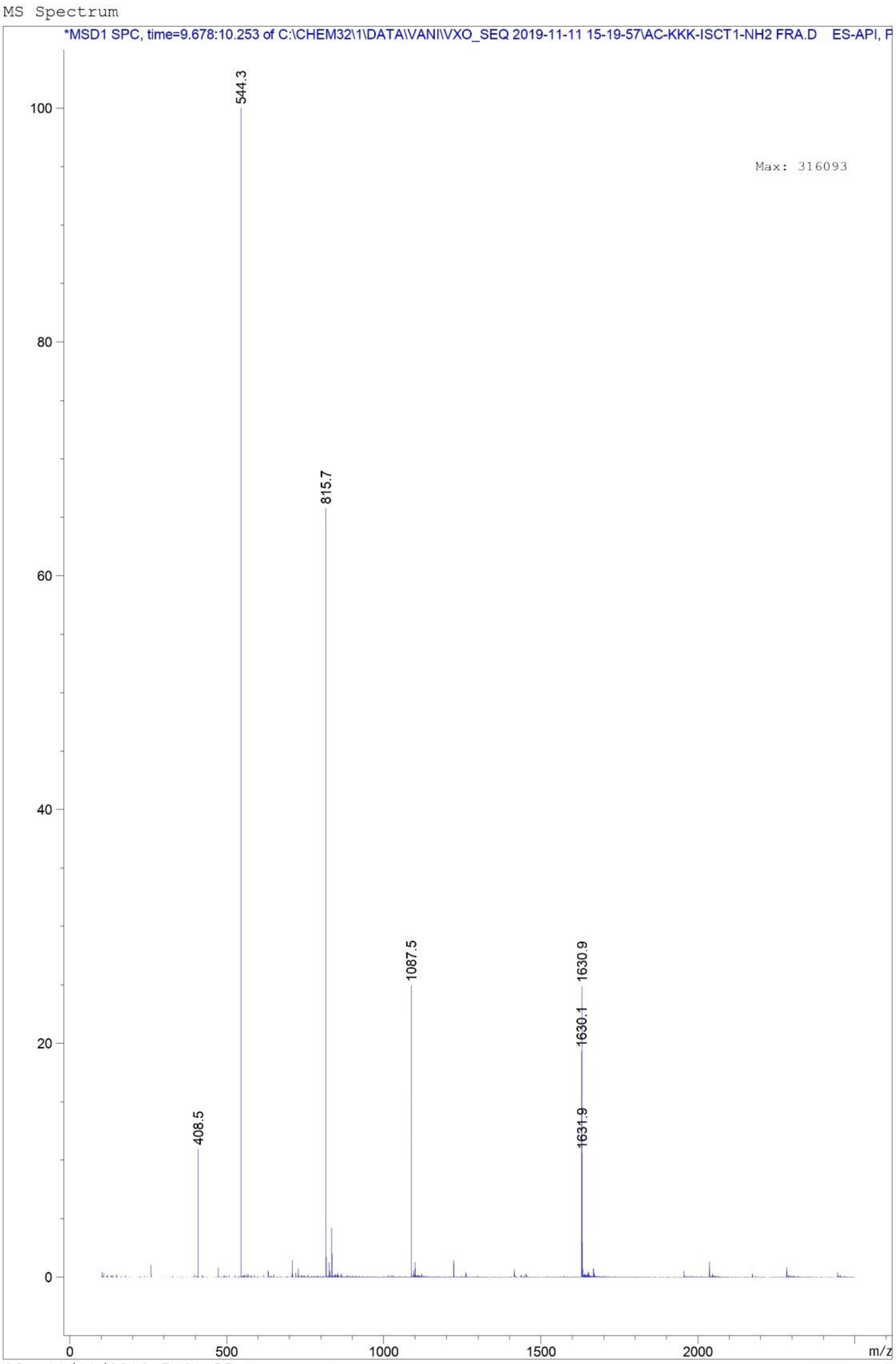

LCMS 6130B 11/11/2019 7:41:57 PM SYSTEM Page 1 of 1

(f)

Peptide	Calculated	Observed mass
IsCT-P	1654,2	1655,0
AFPK-IsCT	1533.9	1535.8
Ac-AFPK-IsCT	1575.9	1577.8
KKK-IsCT	1588.0	1589.9
Ac-KKK-IsCT	1630.1	1631.9

LC/ESI-MS profiles obtained under the following conditions: Column: Column: Phenomenex Gemini C18 (2.0 x 150 mm), 110 Å, 3.0 μm; Solvent system: A: 0.1% TFA/H_2O, B: 90% ACN in A; Gradient: 5-95% B in 30 min; Flow rate: 0.4 mL min-1; λ = 220 nm; Injection volume: 15 μL; Sample concentration: 1.0 mg mL-1 and Mass range: 100–2500 Daltons.

Figure S2:

(a)

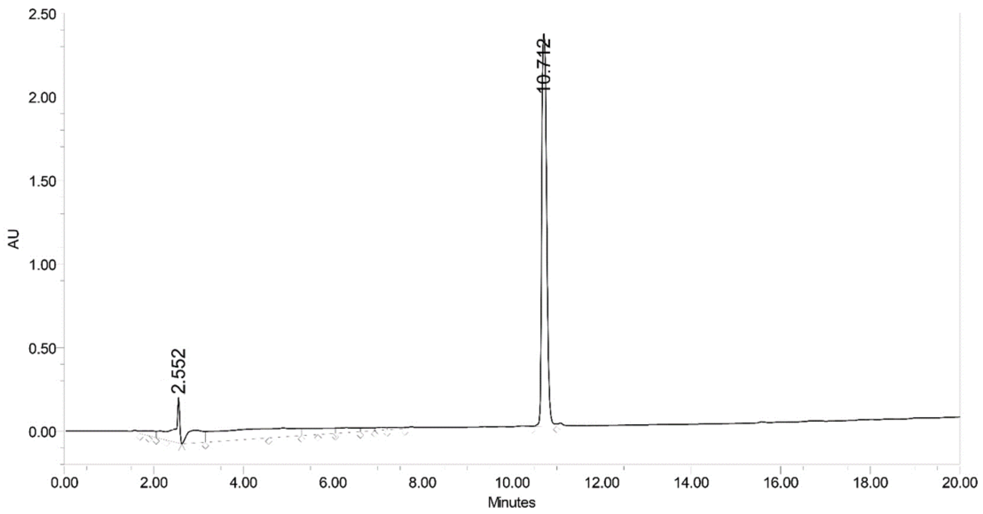

(b)

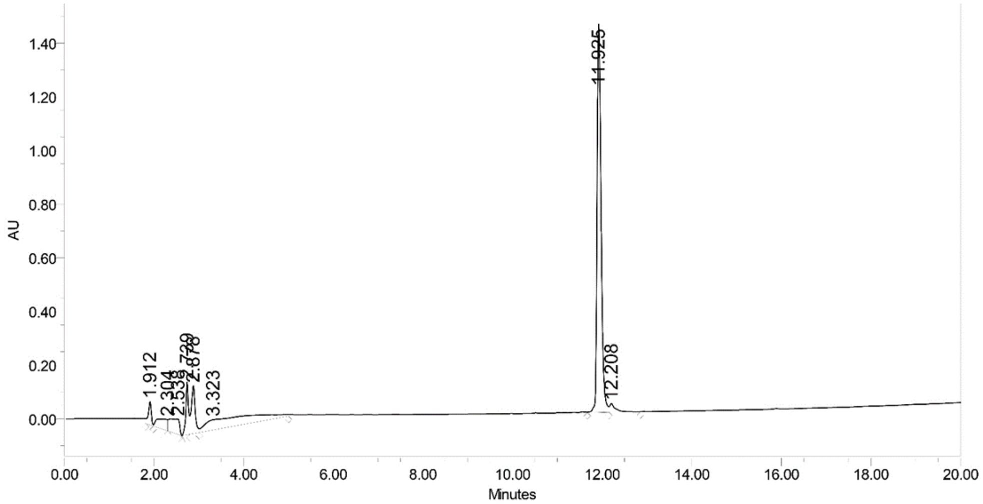

(c)

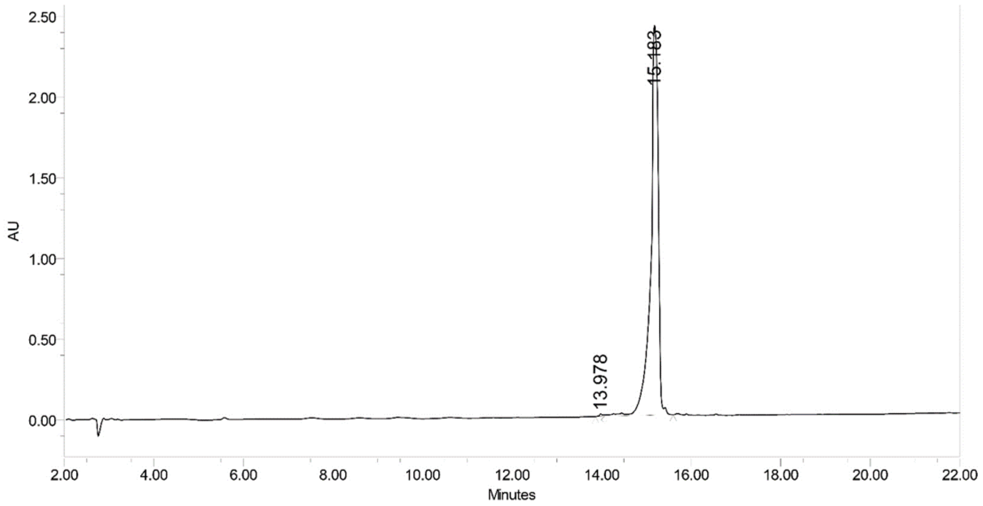

(d)

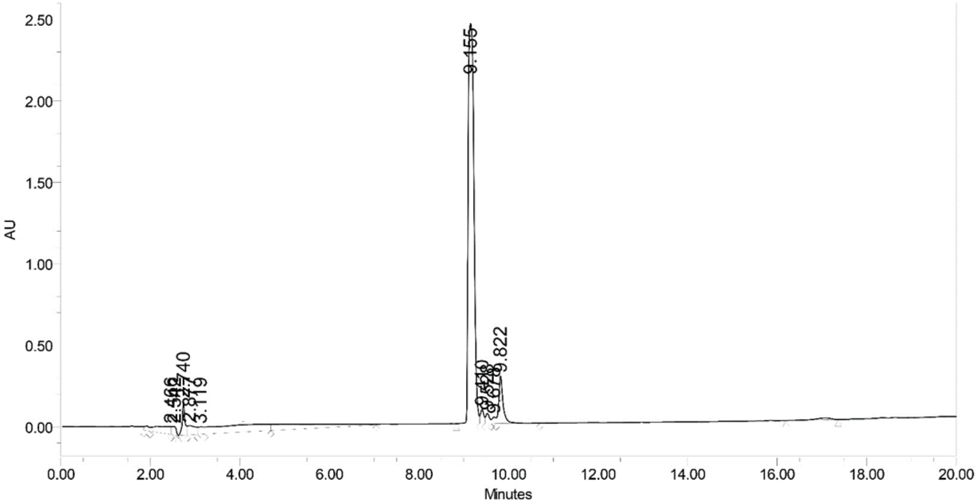

(e)

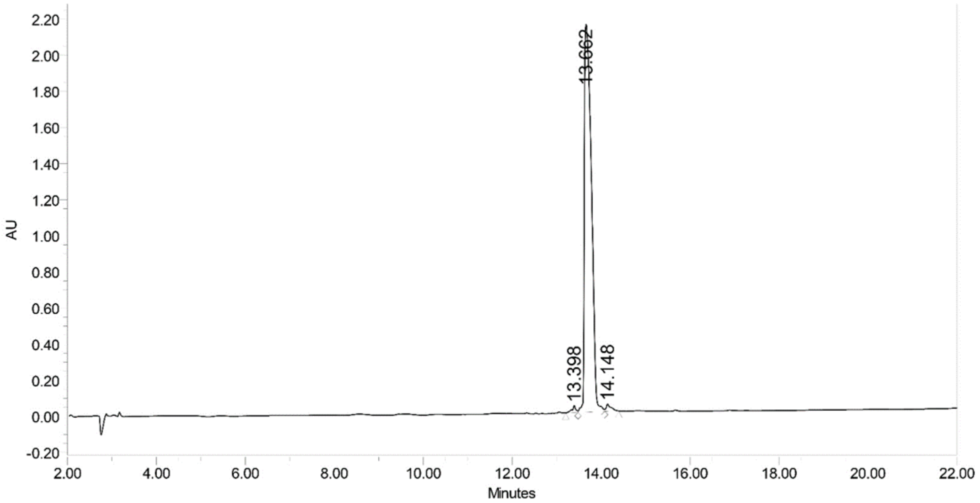

(f)

Peptide	RT	Area (%)
IsCT-P	10.712	99%
AFPK-IsCT	11.925	99%
AC-AFPK-IsCT	15.183	98%
KKK-IsCT	9.156	95%
AC-KKK-IsCT	13.662	97%

HPLC profiles obtained under the following conditions: Column: Supelcosil C18 (4.6 x 150 mm), 60Å, 5 µm; Solvent system: A: 0.1% TFA/H_2O and B: 90% ACN in A; gradient: 5 to 95%B in 20 min; flow rate: 0.8 mL min-1; λ = 220 nm; injection volume: 30 µL and sample concentration: 1.0 mg mL-1.
